# Cholera

**Published:** 1858-07

**Authors:** 


Cholera.

Dr. Pinkerton * thinks the following propositions in relation to the communication of cholera, may be laid down as proved:

1. Cholera spreads *to any considerable distance*—beyond five hundred yards—by personal communication, not by the atmosphere. 2. When once the "cholera poison" is carried to a spot favourable for its development, it will there attach itself, lying dormant for a period of time, not yet fixed by any observation—certainly not less than four months, and is always likely to break out among strangers living over the spot, in a house, hut, or ship. 3. Men so living, fall ill during a time of fatigue, watchfulness, and irregularity of living, as in trench duty, or during an epidemic of diarrhœa.

* The Spread of Cholera by Personal Communication, as seen in the Crimean Campaign. By A. W. P. Pinkerton, M.D.

